# The innervation of the soft palate muscles involved in cleft palate: a review of the literature

**DOI:** 10.1007/s00784-016-1791-6

**Published:** 2016-03-29

**Authors:** Robrecht J. H. Logjes, Ronald L. A. W. Bleys, Corstiaan C. Breugem

**Affiliations:** Division of Plastic and Reconstructive Surgery, University Medical Centre Utrecht, PO Box 85500, 3508 GA Utrecht, The Netherlands; Division of Surgical Specialties, Department of Anatomy, University Medical Centre Utrecht, P.O. Box 85060, 3508 AB Utrecht, The Netherlands; Division of Plastic and Reconstructive Surgery, Wilhelmina Children’s Hospital, PO Box 85090, 3508 AB Utrecht, The Netherlands

**Keywords:** Tensor veli palatini, Levator veli palatini, Palatopharyngeus, Pharyngeal plexus, Glossopharyngeal nerve, Vagus nerve, Lesser palatine nerve

## Abstract

**Objective:**

Surgical techniques to obtain adequate soft palate repair in cleft palate patients elaborate on the muscle repair; however, there is little available information regarding the innervation of muscles. Improved insights into the innervation of the musculature will likely allow improvements in the repair of the cleft palate and subsequently decrease the incidence of velopharyngeal insufficiency. We performed a literature review focusing on recent advances in the understanding of soft palate muscle innervation.

**Material and methods:**

The Medline and Embase databases were searched for anatomical studies concerning the innervation of the soft palate.

**Results:**

Our literature review highlights the lack of accurate information about the innervation of the levator veli palatini and palatopharyngeus muscles. It is probable that the lesser palatine nerve and the pharyngeal plexus dually innervate the levator veli palatini and palatopharyngeus muscles. Nerves of the superior-extravelar part of the levator veli palatini and palatopharyngeus muscles enter the muscle form the lateral side. Subsequently, the lesser palatine nerve enters from the lateral side of the inferior-velar part of the levator veli palatini muscle. This knowledge could aid surgeons during reconstruction of the cleft musculature. The innervation of the tensor veli palatini muscle by a small branch of the mandibular nerve was confirmed in all studies.

**Conclusion:**

Both the levator veli palatini and palatopharyngeus muscles receive motor fibres from the accessory nerve (through the vagus nerve and the glossopharyngeal nerve) and also the lesser palatine nerve. A small branch of the mandibular nerve innervates the tensor veli palatini muscle.

**Clinical relevance:**

Knowledge about these nerves could aid the cleft surgeon to perform a more careful dissection of the lateral side of the musculature.

## Introduction

Achieving adequate velopharyngeal closure for optimal feeding and speech development is a main objective in cleft palate closure. Unfortunately, 20–30 % of the cleft palate closures result in velopharyngeal insufficiency [1, 2]. Numerous surgical techniques for palate closure have been described [3, 4]. Most studies focus on the anatomical repair of the musculature of the cleft palate. Surgical techniques do not mention the possible nerve damage that could result from the surgical dissection. Several studies describing the palatal musculature have been described [5, 6]. However, for an optimal functional muscular repair of the soft palate, thorough understanding of the motor innervation of these soft palate muscles is crucial. This may prevent nerve damage during surgical dissection and therefore may result in a better functional outcome and less complications in patients with cleft palate or velopharyngeal insufficiency. Two major anatomical textbooks mention that both palatopharyngeus muscle (PP) and the levator veli palatini muscle (LVP) are innervated by the cranial part of the accessory nerve (CN XI) via the pharyngeal plexus and the tensor veli palatini muscle (TVP) is innervated by the mandibular nerve [7, 8]. Nevertheless, anatomical uncertainties remain such as the possible involvement of the facial nerve (CN VII) and the exact neural route via the pharyngeal plexus to the soft palate [7]. This review provides an update of our current understanding of the origin, course and ramification patterns of the nerves that supply the three most important soft palate muscles. The muscles discussed are the palatopharyngeus (PP), the levator veli palatini (LVP) and the tensor veli palatini (TVP) muscles. This information could subsequently aid cleft surgeons during the cleft palate repair.

## Methods

An extensive literature search was conducted using Embase and Medline (April 2015) and performed by RJH Logjes. First the terms “soft palate AND (innervation OR nerve)” and “velum AND (innervation OR nerve)” were used. This resulted in respectively *n* = 551 and *n* = 109 results. Secondly, the names of the three muscles were used as a term resulting in “tensor veli palatini” (*n* = 226), “levator veli palatini” (*n* = 283) and “palatopharyngeus” (*n* = 105). After selecting the relevant articles by reading title and abstracts, 12 articles were used in this review. Five articles described the course of the nerves to the soft palate in human cadavers [9–13] and one article described an electromyography (EMG) study [14]. Another article did not describe material and methods [15].

A total of five studies on the innervation of the soft palate muscles in animals were also used in this review [16–20].

## Results

Baseline characteristics of the seven studies on human material and the five studies on animals are presented in Tables 1 and 2.

**Table 1:** Baseline characteristics of the seven studies on human material

Authors	Muscle(s) investigated	*N*	Method	Results
Broomhead [9]	TVP, LVP, PP	1 adult head, 2 foetal heads, 3 human embryos	Dissecting and serial sections after staining HE and Ranson’s silver impregnation	LVP: Pharyngeal plexus of the CN X PP: CN IX and CN X TVP: mandibular nerve (CN V)
Broomhead [10]	TVP, LVP, PP	3 human embryos	Serial sections stained by the De Castro’s method	LVP: Pharyngeal plexus of the CN X PP: CN IX and CN X TVP: mandibular nerve (CN V)
Doménech-Ratto [11]	TVP, LVP, PP	51 embryos	Sectioned transversally, frontally or saggitally and stained by HE (*n* = 35), Azan (*n* = 26) or Bielschowsky method (*n* = 10)	LVP: CN IX PP: CN IX and CN X TVP: mandibular nerve (CN V)
Sedlácková [14]	Soft palate	25 patients with the syndrome of developmental shorting of the soft palate	Electromyography (EMG) of the facial and soft palate muscles	CN VII, CN IX, CN X
Shimokawa et al. [12]	LVP	50 head halves	Dissection by binocular microscope	LVP: 3 types of innervation by pharyngeal plexus (CN IX and X)
Shimokawa et al. [13]	LVP, PP	30 head halves	Dissection by stereomicroscope and staining nerve fibres with silver nitrate as described by Kimura and Takahashi	LVP and PP: Lesser palatine nerve (CN V) and pharyngeal plexus (CN IX and X)
Shankland [15]	TVP	Undescribed	Undescribed	TVP: mandibular nerve (CN V)

*TVP* tensor veli palatini muscle, *LVP* levator veli palatini muscle, *PP* palatopharyngeus muscle, *CN V* trigeminal nerve, *CN VII* facial nerve, *CN IX* glossopharyngeal nerve, *CN X* vagus nerve, *HE* haematoxylin-eosin

**Table 2:** Baseline characteristics of the five studies on animals

Authors	Muscle(s) investigated	*N*	Method	Results
Nishio et al. [16]	LVP, MU, SCP, OO	20 rhesus monkeys	Evoked electromyography (EMG) responses	LVP: CN VII and pharyngeal plexus (CN IX,X)
Ibuki et al. [17]	LVP, OO	10 rhesus monkeys	Evoked electromyography (EMG) responses	LVP: course of the facial nerve for the LVP through the greater petrosal nerve
Van Loveren et al. [18]	LVP	18 cats	HRP injection in LVP and after 24–48 h microscopically examination of the brainstem sections	NA, RFN (CN IX, X) No labelled cells in the FN
Keller et al. [19]	TVP, LVP	19 cats	HRP injection in TVP and LVP and after 24–48 h microscopically examination of the brainstem sections	TVP: TMN LVP: NA, RFN No labelled cells in the FN
Strutz et al. [20]	Soft palate	9 guinea pigs 4 monkeys	HRP injection of the velum and after 48 h microscopically examination of the brainstem sections	NA, RFN (CN IX, X, XI) TMN

*HRP* horseradish peroxidase, *NA* nucleus ambiguus, *RFN* retrofacial nucleus, *TMN* trigeminal motor nucleus, *FN* facial nucleus, *OO* orbicularis oris muscle, *SCP* superior constrictor pharyngeus muscle, *MU* uvulae muscle, *TVP* tensor veli palatini muscle, *LVP* levator veli palatini muscle, *CN VII* facial nerve, *CN IX* glossopharyngeal nerve, *CN X* vagus nerve, *CN XI* accessory nerve

### Levator veli palatini

Most authors agree that the LVP is supplied by the pharyngeal plexus [9, 10, 12–14]. However, minor differences in description exist. Broomhead claims that the pharyngeal plexus contains branches of the vagus nerve only, while other authors state that this plexus receives contributions from the glossopharyngeal and vagus nerves [9, 10, 12–14]. Doménech-Ratto found that the glossopharyngeal nerve reached the LVP without forming a plexus [11]. Sedlácková et al. concluded from their EMG records in 25 patients that facial muscle disorders combine with a disorder of the LVP and that therefore the LVP is dually innervated: via the facial nerve during speech and via the pharyngeal plexus (the glossopharyngeal and vagus nerves) during swallowing [14]. A dual innervation of the LVP was also found by Shimokawa et al. who claims that the lesser palatine nerve and the pharyngeal plexus are innervating the LVP [13].

Four studies showed more insight into morphological details of the pharyngeal plexus and the course of the supplying nerve towards the LVP. The contributions to the pharyngeal plexus from the glossopharyngeal and vagus nerves may run as a joint nerve along carotid artery branches or may form a true plexus with multiple communications, together with the branches of the sympathetic trunk in their inferomedial course between the internal and external carotid arteries [9, 10, 12, 13].

Broomhead states in two articles that one pharyngeal plexus branch, derived from the vagus nerve, ascends vertically on the lateral side of the constrictor muscles. This nerve branch passes forwards across the sinus of Morgagni at the level of the upper border of the superior constrictor muscle. It divides into two smaller branches before entering the lower lateral border of the extravelar muscle part of the LVP [9, 10].

**Fig. 1:**
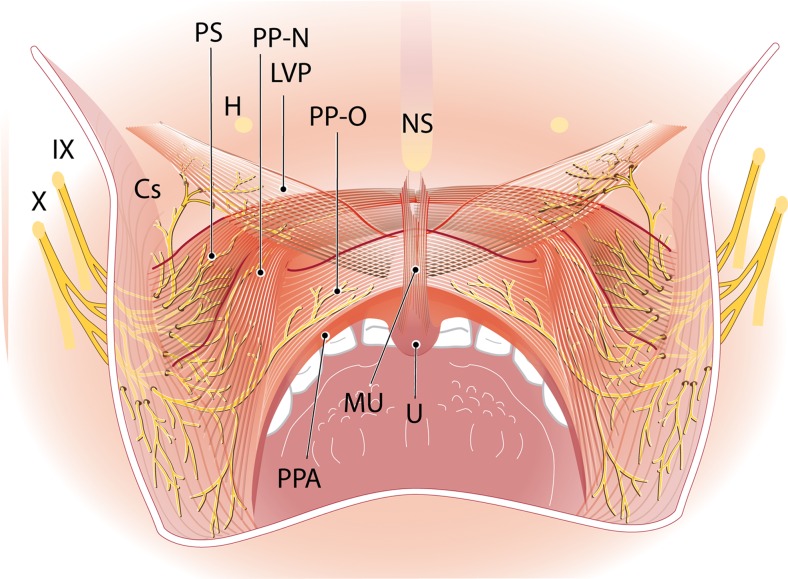
Adapted from Shimokawa et al. [13] by I. Janssen. Dorsal view. Distribution of the pharyngeal plexus in the superior-extravelar part of the LVP and nasal and oral parts of the PP. *LVP* levator veli palatini muscle, *PP-N* palatopharyngeus muscle nasal strand, *PP-O* palatopharyngeus muscle oral strand, *PS* palatopharyngeal sphincter, *MU* uvulae muscle, *PPA* palatopharyngeal arch, *Cs* constrictor superior muscle, *H* hamulus, *NS* nasal septum, *U* uvula, *IX* glossopharyngeal nerve, *X* vagus nerve

According to Broomhead, small branches of the ascending pharyngeal artery accompany this nerve branch on the surface of the constrictor muscles [9]. According to Shimokawa et al. the LVP branch always has a common trunk with the supplying branch to the superior constrictor muscle. It penetrates the superior constrictor first and subsequently enters the LVP at its posterior margin. Tiny branches are distributed throughout the superior-extravelar muscle part of the LVP [12, 13] (Fig. 1). This superior-extravelar muscle part of the LVP, which is supplied by the pharyngeal plexus, is much bigger than the inferior-velar part of the LVP which, according to Shimokawa et al., is supplied by the lesser palatine nerve [13]. Shimokawa et al. distinguished three patterns of innervation of the superior part of the LVP, based on their origin in the pharyngeal plexus. Type 1: the supplying nerve branches find their origin in the glossopharyngeal nerve only. Type 2: the supplying nerve branches originate from communicating branches between the glossopharyngeal and vagus nerves. Type 3: the supplying nerve branches find their origin in the vagus nerve only. Type 2 innervation pattern was found in most specimens in a human cadaver study done by Shimokawa et al. [12].

**Fig. 2:**
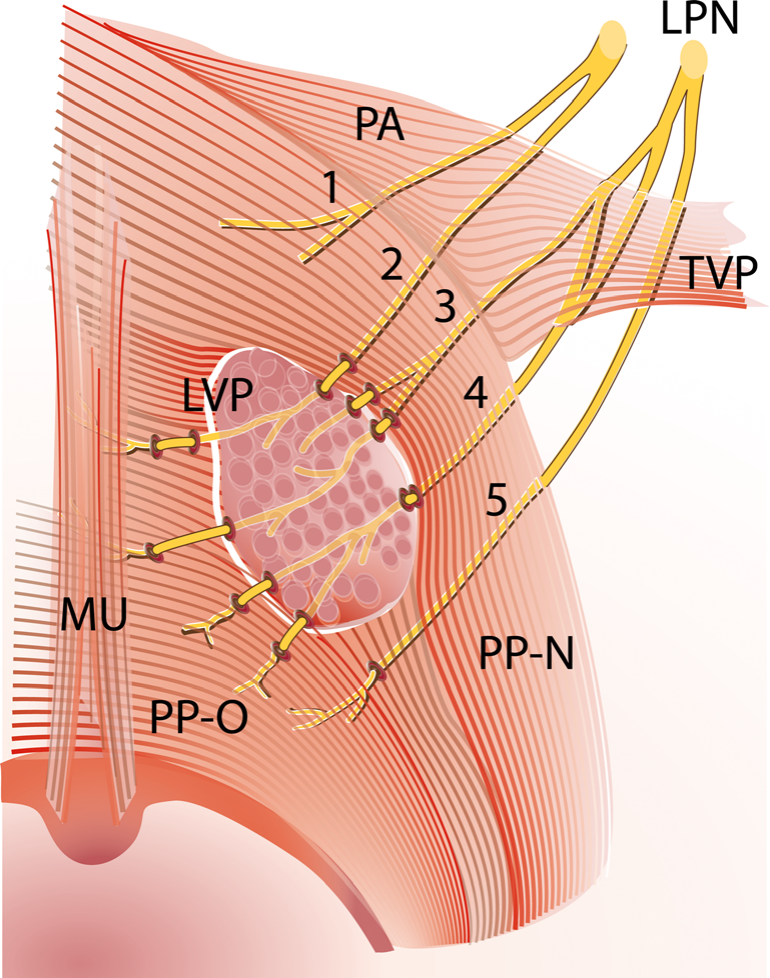
Superior view on soft palate with the innervation by the lesser palatine nerve, adapted from Shimokawa et al. [13] by I. Janssen. A part of the LVP is removed for better view on the five nerve fibres of the lesser palatine nerve, which were found in the human cadaver study by Shimokawa et al. [13]. These nerves run underneath the palatine aponeurosis and the nasal part of the PP and penetrate the inferior-velar part of the LVP on its lateral surface close to the insertion of the LVP in the midline of the velum. *LPN* lesser palatine nerve, *LVP* levator veli palatini muscle, *PA* palatine aponeurosis, *PP-N* palatopharyngeus muscle nasal strand, *PP-O* palatopharyngeus muscle oral strand, *TVP* tensor veli palatini muscle, *MU* uvulae muscle

A contribution from the lesser palatine nerve runs through the lesser palatine foramen and ramifies in multiple small nerve branches. These branches run posteromedially underneath the palatine aponeurosis and the nasal part of the PP. Close to the insertion of the LVP in the midline of the velum, almost all branches enter the muscle on its lateral surface. The small nerve branches end up in the inferior-velar part of the LVP and the bigger nerve branches penetrate this muscle to end up in the uvulae muscle or the oral part of the PP (Fig. 2). Most of the time, the anteriormost branch of the lesser palatine nerve does not penetrate the LVP but runs underneath the palatine aponeurosis and ends in the glandular tissue of the palate [13]. In three specimens, Shimokawa et al. found a variation where the posteriormost branch of the lesser palatine nerve enters the PP directly without penetrating the LVP. This entering point on the inferior surface of the medial part of the PP is close to the posterior border of the LVP [13] (Fig. 2).

### Palatopharyngeus

There is agreement that the palatopharyngeus is supplied by the pharyngeal plexus through contributions from the glossopharyngeal and vagus nerves [9–11, 13]. According to Broomhead, the pharyngeal branch of the vagus nerve reaches the lower part of the PP, after running parallel to the posterior border of stylopharyngeus and entering the pharynx between superior and middle constrictors. The glossopharyngeal nerve branch enters the PP, after a course along the anterior border of the stylopharyngeus and coursing between the same constrictors. Once these branches enter the PP, they divide into very small nerve branches between the muscle fibres [9]. According to Shimokawa et al., there is a minor additional supply from the lesser palatine nerve. This branch supplies the anterior part of the oral part of PP. The same authors state that the PP branch from the pharyngeal plexus is from a common branch with the supplying nerves of superior and middle constrictors. After penetrating the constrictors or running between these muscles, the nerve enters the PP on its lateral surface. Smaller nerve branches ascend inside the PP and are distributed in the oral and nasal parts of the muscle [13] (Fig. 1).

Summarizing, all the authors who investigated the human nerve supply towards the soft palate agree that the pharyngeal plexus innervates the levator veli palatini and the palatopharyngeus muscle [9–14]. Although the descriptions of the exact composition of the pharyngeal plexus vary among the authors. According to Broomhead, the pharyngeal plexus differs in innervating these two muscles: towards LVP it contains only the vagus nerve; towards the PP it contains both the glossopharyngeal and vagus nerves [9, 10]. On the contrary, Doménech-Ratto claims that only the glossopharyngeal nerve innervates the LVP and agrees with Broomhead that the glossopharyngeal and vagus nerves together innervate the PP [11].

Sedlácková et al. think the LVP is innervated via the facial nerve during speech and via the pharyngeal plexus, which contains the glossopharyngeal and vagus nerves, during swallowing [14].

**Fig. 3:**
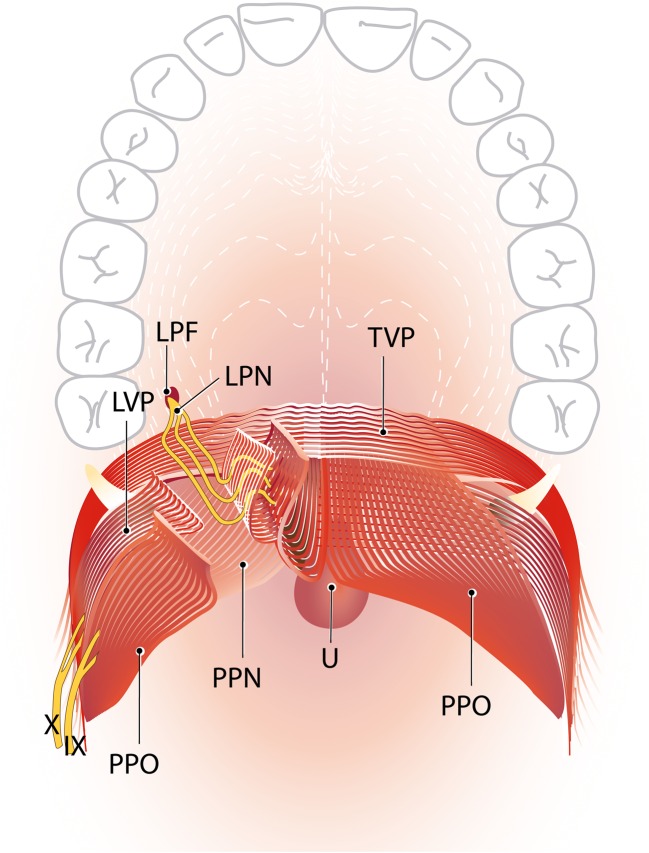
The dual innervation of the soft palate by the LPN and the pharyngeal plexus (idea and design by RJH Logjes and CC Breugem after combining the two innervation patterns shown in Figs. 1 and 2, illustrated by I Janssen). View of the plastic surgeon on the soft palate; the pharyngeal plexus penetrates the superior-extravelar part of the LVP on the lower lateral border. The lesser palatine nerve runs through the lesser palatine foramen and runs over the palatine aponeurosis of the TVP and the nasal part of the PP to enter the inferior-velar part of the LVP on its lateral surface. Here, the LPN innervates the small inferior-velar part of the LVP and the anterior part of the oral part of the PP, together referred to as the anteromedial region of the soft palate muscles. *LPF* lesser palatine foramen, *LPN* lesser palatine nerve, *IX* glossopharyngeal nerve, *X* vagus nerve, *TVP* tensor veli palatine muscle, *LVP* levator veli palatini muscle, *PP-N* palatopharyngeus muscle nasal strand, *PP-O* palatopharyngeus muscle oral strand, *U* uvula

Shimokawa et al. concluded that both the lesser palatine nerve and the pharyngeal plexus, which contains the glossopharyngeal and vagus nerves, dually innervate the soft palate muscles LVP and PP. The lesser palatine nerve innervates the small inferior-velar part of the LVP and the anterior part of the oral part of the PP, together referred to as the anteromedial region of the soft palate muscles. The pharyngeal plexus innervates the bigger superior part of the LVP and the nasal and remaining oral part of the PP, also referred to as the posterolateral region of the soft palate muscles [13]. Figure 3 demonstrates the view of the cleft surgeon on the soft palate and the course of both the lesser palatine nerve as the pharyngeal plexus.

### Tensor veli palatini

There is an agreement that the TVP is supplied by the mandibular nerve, which is the third branch of the trigeminal nerve [9–11, 15]. According to Shankland, the mandibular nerve splits into an anterior and a posterior part in the infratemporal fossa just after its passage through the foramen ovale. Just before this division, a small branch is given off, the so-called undivided trunk of the mandibular nerve. This undivided trunk ramifies into four smaller nerve branches. One of these smaller branches is the nerve to the TVP. This nerve runs through the otic ganglion without having a functional relationship with it and then enters the TVP close to its origin in the scaphoid fossa of the sphenoid bone. The other three branches of this undivided trunk include the nerve to medial pterygoid, nerve to tensor tympani and the meningeal branch also known as nervus spinosus [15]. Broomhead found the supplying branch to the TVP splitting from the nerve to medial pterygoid, passing forwards and medially to enter the posterior border of TVP as one branch or the lateral and posterior borders via two branches [9, 10].

### Nerve supply in animals

Van Loveren et al., Keller et al. and Strutz et al. localized motorneurons of the soft palate muscles in the brainstem of cats, guinea pigs and monkeys by using a retrograde neuroanatomical tracing technique [18–20]. Nishio et al. and Ibuki et al. studied the motor innervation of respectively the soft palate muscles and the LVP by evoked electromyography (EMG) responses in rhesus monkeys [16, 17].

Except for the TVP, there is controversy about the location of the motorneurons for the soft palate muscles. The TVP in humans receives its motor fibres from the trigeminal nerve [9, 11, 15]. Keller et al. confirmed this by retrograde tracing [19].

Nishio et al. claims that the soft palate receives motor fibres from both the facial nerve and pharyngeal plexus of the glossopharyngeal and vagus nerves [16]. Ibuki et al. found that the LVP was only supplied by the facial nerve [17]. Both Nishio et al. and Ibuki et al. agreed that motor fibres of the facial nerve ran through the pterygopalatine ganglion and mixed with sensory fibres of the lesser palatine nerve to reach the soft palate and the LVP, respectively [16, 17]. On the other hand, Van Loveren et al. and Keller et al. excluded the role of the facial nerve in the motor innervation of the LVP and found the LVP dually motor innervated by the glossopharyngeal and vagus nerves [18, 19]. Strutz et al. claimed that the glossopharyngeal, vagus and accessory nerves are all responsible for the motor supply of the soft palate muscles [20].

## Discussion

Only few studies investigated the innervation of the soft palate muscles in humans. The innervation of the TVP by the mandibular nerve is universally accepted [9–11, 15]. However, knowledge about the innervation of the LVP and PP remains controversial. All authors mentioned the contribution of the pharyngeal plexus but the details of their descriptions vary. According to two major anatomical textbooks, the pharyngeal plexus receives its motor fibres from the cranial part of the accessory nerve [7, 8].

The studies by Broomhead were on small series of human heads [9, 10]. The same applies to the study by Doménech-Ratto who only investigated 10 embryos [11]. Studies by Shimokawa et al. were far more extensive and resulted in more robust conclusions about the course of the nerves and ramification patterns towards the soft palate muscles [12, 13]. Shimokawa et al. in 2004, who mainly focussed on the LVP and the superior constrictor, did not report any contribution of the lesser palatine nerve to the supply of LVP [12]. In another manuscript, Shimokawa et al. concluded in 2005 that the LVP and PP are innervated by the lesser palatine nerve and the pharyngeal plexus. Subsequently, in this second manuscript, nerve staining was performed by Shimokawa et al., which could explain the finding of the small lesser palatine nerve innervating part of the soft palate [13].

Furthermore, Shimokawa et al. assumes that the hypothesis of Nishio et al. and Ibuki et al. that motor fibres of the facial nerve run inside the lesser palatine nerve in animals is also applicable to humans [13, 16, 17]. Shimokawa et al. dissected the lesser palatine nerve but did not perform a functional characterization of the nerve fibres [13]. Gray’s Anatomy states that every branch of the trigeminal nerve contains afferent fibres, including the maxillary nerve from which the lesser palatine nerve is derived [8]. There is a possibility that lesser palatine nerve fibres which run to the LVP and PP contain sensory fibres only, namely for propriocepsis, pain and temperature information. There are examples in human anatomy where motor and sensory supplies of muscles go via different nerves. For instance, the trapezius muscle receives its motor supply from the spinal root of the accessory nerve and plexus cervicalis, whereas only the second, third and fourth cervical spinal nerves carry proprioceptive fibres from it [21]. Another example are the facial muscles, which are efferently innervated by the facial nerve while their afferent fibres are part of the trigeminal nerve and end up in the mesencephalic nucleus [8].

It would be useful to investigate the presence of motor fibres in the lesser palatine nerve by specific staining techniques. Sedlácková et al. did the only human study that assumes the involvement of the facial nerve innervating the LVP together with the pharyngeal plexus [14].

Studies on the motor nerves to the soft palate muscles in animals had very conflicting results and seem to be less useful as a model for the human situation.

This review demonstrates crucial information for the cleft surgeon that innervation of the superior-extravelar part of the LVP and the PP enters the muscle form the lateral side. Subsequently, the lesser palatine nerve enters from the lateral side of the inferior-velar nasal part of the LVP. Although this anatomy is applicable to the normal soft palate, it will likely be applicable to the cleft palate. During cleft surgery, intravelar velar reposition is performed when the LVP is released from PP and retropostioned ventrally [4]. This analysis suggests that during surgical dissection caution should be taken to dissect the dorsal/lateral aspect of the LVP from the PP because that is the area where the lesser palatine nerve enters the LVP. This theory is applicable to the von Langenbeck, two flap palatoplasty and also to the Furlow plasty. During the von Langenbeck procedure, the LVP should be adequately released from the nasal mucosa (and a thin layer of PP) and care should be taken not to perform a rigorous dissection on the lateral side of the LVP.

## Conclusion

This review of the literature demonstrates the lack of accurate information about the innervation of the levator veli palatini and palatopharyngeus muscle. Most likely, the lesser palatine nerve and the pharyngeal plexus dually innervate these two muscles. However, since the type of nerve fibres of the lesser palatine nerve is unclear, the role of the facial nerve in motor-innervating the soft palate is uncertain. The pharyngeal plexus plays a major role in innervating the levator veli palatini and palatopharyngeus muscle and receive its motor fibres from the accessory nerve. The tensor veli palatini is innervated by the mandibular nerve. This information should aid the surgeon during repair of the cleft palate.
